# A Game-Based Approach to Lower Blood Pressure? Comparing Acute Hemodynamic Responses to Endurance Exercise and Exergaming: A Randomized Crossover Trial

**DOI:** 10.3390/ijerph19031349

**Published:** 2022-01-26

**Authors:** Eva Kircher, Sascha Ketelhut, Kerstin Ketelhut, Lisa Röglin, Kuno Hottenrott, Anna Lisa Martin-Niedecken, Reinhard G. Ketelhut

**Affiliations:** 1 Department of Medical Sciences, Charité University Medicine Berlin, 10117 Berlin, Germany; eva.kircher@t-online.de (E.K.); r.ketelhut@t-online.de (R.G.K.); 2 Institute of Sport Science, Martin Luther University Halle-Wittenberg, 06120 Halle (Saale), Germany; li.roeglin@googlemail.com (L.R.); kuno.hottenrott@sport.uni-halle.de (K.H.); 3 Faculty of Natural Science, MSB Medical School Berlin, 14197 Berlin, Germany; kerstin.ketelhut@medicalschool-berlin.de; 4 Department of Design, Institute for Design Research, Zurich University of the Arts, 8031 Zurich, Switzerland; anna.martin@zhdk.ch; 5 Cardiology and Sports Medicine, Medical Center Berlin (MCB), 10559 Berlin, Germany

**Keywords:** exergaming, hemodynamics, peripheral blood pressure, central blood pressure, post-exercise hypotension, moderate endurance training

## Abstract

The present randomized crossover study aimed to determine whether an exergaming session in an innovative, functional fitness game could be an effective exercise approach that elicits favorable blood pressure (BP) responses, such as a typical moderate endurance exercise (ET). Therefore, acute hemodynamic responses after a training session in the ExerCube and an ET on a treadmill were assessed and compared. Twenty-eight healthy recreational active participants (13 women; aged 24.8 ± 3.9 years) completed an exergaming session (EX) and an ET in a randomized and counterbalanced order. Before and throughout the 45 min after the training, the peripheral and central BP were measured. After the ET, there was a moderate decrease in both peripheral systolic (−1.8 mmHg; *p* = 0.14) and diastolic (−0.8 mmHg; *p* = 0.003), as well as central diastolic (−1.5 mmHg; *p* = 0.006) pressure compared to the resting value before the exercise. After the EX, there was a significant decrease in peripheral systolic (−6.3 mmHg; *p* < 0.001) and diastolic (−4.8 mmHg; *p* < 0.001), as well as central systolic (−5.8 mmHg; *p* < 0.001) and diastolic (−5.3 mmHg; *p* < 0.001) pressure compared to baseline. The interaction effects showed significant differences in peripheral and central systolic BP as well as in peripheral diastolic BP (*p* = 0.05). The EX seems to be an effective training approach that triggers relevant peripheral and central BP-responses, which are more pronounced than after a typical ET. Therefore, the ExerCube can be a time-efficient training tool to improve cardiovascular health.

## 1. Introduction

Hypertension, which is often caused by a lack of physical activity (PA), is a leading cause of cardiovascular diseases, reduced quality of life, and premature death. It is estimated that 7.5 million deaths each year around the world are attributed to high blood pressure [1].

Regular aerobic exercise, regarded as a key lifestyle intervention for both the treatment and prevention of hypertension, has been proven to reduce both systolic (SBP) and diastolic (DBP) blood pressure (BP) in various populations [2]. Apart from regular aerobic exercise, an acute bout of exercise already leads to a temporary lowering of BP [3,4]. The immediate reduction in BP after a single exercise session is termed as post-exercise hypotension (PEH). It is expected that the summation of the BP reductions that occur during the immediate recovery periods is responsible for long-term BP reductions [5]. Hence, PEH helps predict the BP response to regular exercise and thus the effectiveness of BP-related exercise training [3,4].

Although the positive cardiovascular effects of regular exercise are widely recognized, more than a quarter of adults worldwide do not meet the PA recommendation of ≥150 min/week of moderate-intensity PA or ≥75 min/week of vigorous-intensity PA [6], which puts them at higher risk for developing hypertension. Even though adults’ participation in regular exercise is influenced by different personal, social, and environmental factors, a lack of motivation is reported to be one of the most common barriers [7]. In this context, exergames have developed as a promising approach to increase PA levels and enable a joyful exercise experience.

Exergames are interactive video games that encourage an active gaming experience. By combining electronic entertainment with physical exercise, exergames create novel opportunities to expand PA in different age groups and settings. Due to their playful and motivational nature, exergames may present an attractive alternative or addition to traditional exercise modes [8].

Even though scholars have found an increase in energy expenditure when playing exergames compared to resting and inactive (sedentary) videogames [9], most games only induce low to moderate-intensity activities that are claimed to be too low to produce relevant physical adaptations and health-related outcomes [10]. This may be due to the fact that most exergames fail to feature both an attractive game design and an effective training concept [11]. It is therefore questionable whether current exergames represent a suitable and effective training approach for cardiovascular prevention.

Recently, a new functional fitness game setting called ExerCube came on the market. The ExerCube promises an individually tailored fitness game that combines innovative and motivating software and hardware design with a holistic training concept [12,13].

The present study aimed to determine whether the ExerCube is an effective training approach that triggers relevant BP responses, such as conventional endurance exercise. Therefore, the acute hemodynamic responses of a single exergaming session (EX) in the ExerCube were compared with PEH effects after a moderate endurance training (ET) on a treadmill. In addition to peripheral BP, central BP was also assessed as it has a significantly higher predictive value with regard to future cardiovascular morbidity and mortality [14].

We hypothesized that the EX could induce similar acute reductions in peripheral and central BP as a single ET.

## 2. Materials and Methods

### 2.1. Participants

Twenty-eight healthy, recreational active participants (46.4% female; aged 24.8 ± 3.9 years; BMI 23.2 ± 2.3 kg/m^2^) volunteered to take part in this study.

Entry criteria included healthy adults (>18 years) of either sex. Participants were excluded from the study if they (1) used antihypertensive or other cardiovascular medications or had previously been treated with cardiovascular drugs; (2) suffered from known cardiovascular diseases, (3) or orthopedic injuries; and (4) had previous experience with the ExerCube. All female participants had to have regular and healthy menstrual cycles, no reported history of menstrual distress, and have utilized an oral contraceptive pill for the previous six months or longer.

Participants were recruited via word of mouth, social media, and posters and flyers displayed at the university campus between October 2019 and December 2019. The participants were informed about the content and structure of the study and gave written consent before the start of the study.

The study was conducted in accordance with the Helsinki Declaration and approved by the Research Ethics Board of the Martin-Luther-Universität Halle-Wittenberg (Medical Faculty of the Martin-Luther-University Halle-Wittenberg 2019-177). The study was pre-registered at ISRCTN registry (ISRCTN43067716, 38154).

### 2.2. Study Design

This study is a randomized crossover trial. Participants reported to the laboratory of the Institute of Sport Science at the Martin Luther University Halle-Wittenberg between December 2019 and May 2020 on three occasions in a 4-hour postprandial state. They were instructed to abstain from consuming caffeinated or alcoholic beverages and nicotine for 4 h. They were further asked to abstain from intensive physical exercise for at least 12 h. All visits were held at least 48 h apart, with each visit occurring at approximately the same time of day. For female participants, the examination days were selected to not fall into the early follicular phase, where the post-exercise hypotension effect is supposed to be more pronounced [15].

During the first visit, participants completed baseline surveys, anthropometry, BP readings, and a graded exercise test (GXT). They were then familiarized with the ExerCube and had the opportunity to participate in a 10-min tutorial.

On the second and third visits, participants completed an ET on a treadmill and an EX in a randomized and balanced order. The principal investigator carried out the randomization using a computerized random number generator. Before and during a 45-min resting phase after the respective exercise session, hemodynamic measurements were obtained.

The same trained study staff member undertook all measurements in the same temperature-controlled laboratory (23.5 °C ± 0.5 °C).

Due to the intervention design, both patients and study staff were not blinded. Only the statistician was blinded to coding of group allocation. The CONSORT flow diagram and checklist are available in the appendix.

### 2.3. Baseline Assessments

Standing height was measured barefoot to the nearest 0.5 cm using a wall-mounted anthropometer. Body mass was assessed to the nearest 0.1 kg using an electronic scale (BC-545 Innerscan, Tanita, Amsterdam, The Netherlands). Waist circumference was measured midway between the lowest ribs and the iliac crest to the nearest 0.1 cm using a nonelastic anthropometric tape.

Participants completed baseline questionnaires assessing habitual physical activity and medical history. Furthermore, a blood pressure measurement was performed to familiarize participants with the procedure.

### 2.4. Incremental Exercise Test

Participants completed a GXT on a treadmill (h/p/cosmos, Pulsar 4.0, Nussdorf—Traunstein, Germany) till voluntary exertion. The initial speed was set according to the individual training status ranging between 7.5 km/h and 10.5 kmh. Each step lasted 3 min interspersed with a 1-min passive rest to draw lactate samples (10 µL) from the earlobe. After each step, the speed was increased by 1.5 km/h until volitional exhaustion.

Heart rate (HR) was monitored throughout the test using a Polar heart rate monitor (Polar Electro OY, Kempele, Finland), and HRpeak was the highest recorded value.

Blood lactate concentrations were assessed at the end of each stage using the enzymatic amperometry method (Dr. Mueller, Super GL ambulance, Freital, Germany). Collected data were processed utilizing WinLactat 3.1 software (Mesics, Münster, Germany), and individual thresholds were derived from the lactate-velocity curve using the Dickhuth model [16].

### 2.5. Hemodynamic Measurements

Before each exercise session, resting hemodynamic parameters, such as the peripheral systolic and diastolic as well as the central systolic and diastolic BP, were measured using the Mobil-O-Graph (24 PWA monitor, IEM, Stolberg, Germany) as a clinically validated device for hemodynamic measurements [17] with a novel transfer function-like algorithm, using brachial cuff-based waveform recordings. Measurements were obtained after a 15-min resting phase in the supine position. A minimum of two readings was taken from the right arm using custom-fit arm cuffs.

Hemodynamic measurements were repeated following both exercise sessions after 15 min, 30 min, and 45 min of rest in a supine position. All measurements were carried out in a quiet environment by the same qualified person in a separate room at controlled room temperature.

### 2.6. ExerCube

The ExerCube is a physically and cognitively challenging exergame where the players are surrounded by three walls, which serve as projection screens and a haptic interface for bodily interactions. A customized motion tracking system tracks players’ movement via HTC Vive trackers (attached to their wrists and ankles). To ensure an attractive and effective workout experience for a broad spectrum of players with different skill levels, the ExerCube continuously adapts game difficulty to players’ fitness and cognitive skills [13].

In this study, the participants played the game Sphery Racer, a single-player game for the ExerCube setting. In this game, the player navigates an avatar on a hoverboard along a racing track and performs different movement tasks (e.g., squats, launches, punches, burpees). The game implements five dynamic movement levels (level 1: 2.5 min; level 2: 2.5 min; level 3: 5 min; level 4: 5 min; level 5: 10 min), which gradually increase in difficulty and complexity. The different levels are interspersed by short (≈30 s) rest periods. Throughout the game, both in-game performance and HR are monitored. To guarantee an optimal cognitive and physical stimulus, game difficulty and speed are adjusted accordingly. Whenever participants accumulate too many mistakes or reach a predetermined HR, the game’s speed decreases. A more extensive description can be found in Martin-Niedecken et al. [12,13].

To determine fluid loss, body mass was assessed to the nearest 0.1 kg using an electronic scale (BC-545 Innerscan, Tanita, The Netherlands) before and after the exercise session in the ExerCube. Throughout the exercise session, HR was continuously recorded using a Polar HR monitor (Polar Electro OY, Kempele, Finland).

### 2.7. Moderate Endurance Exercise

The ET consisted of a 35-min moderate endurance exercise on a treadmill. After a 5-min warm-up (5.5 kmh), treadmill speed was set according to the individual aerobic threshold and continuously adjusted so that each participant attained an HR of <65% of HRpeak throughout the exercise session. HR was monitored by a Polar HR monitor (Polar Electro OY, Kempele, Finland). Before and after the exercise, body mass was assessed to the nearest 0.1 kg using an electronic scale (BC-545 Innerscan, Tanita, The Netherlands).

### 2.8. Statistical Analysis

An a priori power analysis utilizing G*power (Version 3.1.2; Heinrich Heine Universität, Dusseldorf, Germany) was conducted, indicating that a sample size of 24 subjects would provide sufficient power to observe differences, assuming a large effect size. Analysis of the anthropometric data of the participants was carried out using Microsoft Excel 2013. Results are presented as mean and standard deviation. All other statistical analyses were performed using the open-source program “R” (RStudio, Inc., Boston, MA, USA, Version 3.5.3). The Shapiro–Wilk test and the Kolmogorov–Smirnov test were performed to verify the normal distribution of the data. Due to the non-symmetrically distributed samples, a two-sided Wilcoxon test was used for connected samples. The variables are shown as the median and first and third quartile. The level of significance was set at *p* < 0.05. 

## 3. Results

All twenty-eight participants completed both sessions with no adverse events and were analyzed for the primary outcome. Participants’ characteristics are presented in Table 1. On average, the participants were engaged in 7.1 ± 3.4 h of physical exercise/sports per week. According to BMI, four participants were classified as overweight [18]. By means of the waist-to-height ratio (WHtR), two of the included participants showed values within the overweight range. According to the BP classification of the European Society of Cardiology (ESC) [19], none of the participants enrolled could be classified as hypertensive concerning SBP. Two of the participants presented a high normal SBP. According to the DBP, all participants were classified as normotensive.

During the EX, the participants reached a maximal HR of 187.4 ± 9.2 bpm, which corresponds to 96.6 ± 3.6% of their individual HRpeak. The mean HR throughout the game (including the short rest periods between the levels) was 167.1 ± 10.9 bpm, corresponding to 86.1 ± 4.3% of HRpeak. This is significantly (*p* < 0.001) higher than the mean and maximum HR during the ET (138.0 ± 7.7; 152.1 ± 9.6 bpm).

After the ET (45 min), there was only a slight, however, statistically significant reduction in the DBP (Table 2). After the EX, both SBP and the DBP were still significantly reduced 45 min after cessation of the training (Table 2).

**Figure 1:**
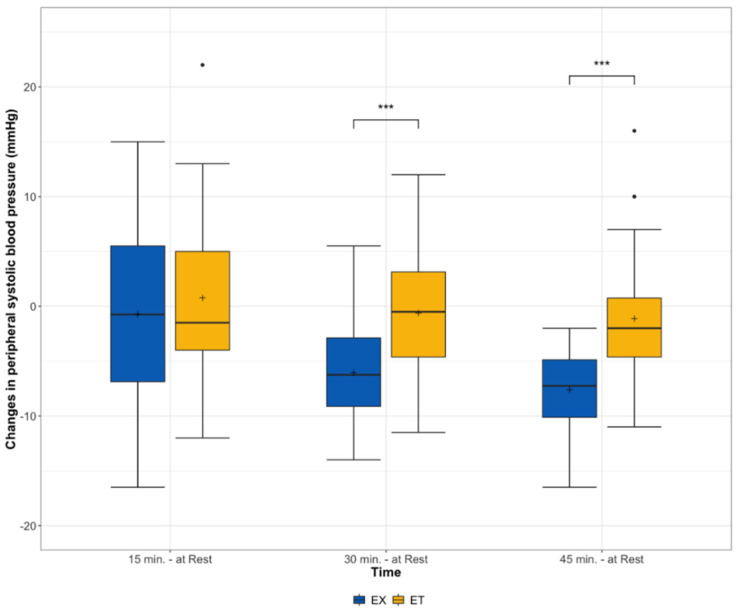
Changes in peripheral systolic blood pressure from rest before and 15, 30, and 45 min after exercise stratified according to the type of exercise (ET = moderate endurance exercise, EX = exergaming session), interaction effect *** = *p* < 0.001.

In peripheral SBP, the interaction effects between both groups showed significant differences in favor of the EX 30 min (*p* < 0.001) as well as 45 min (*p* < 0.001) after exercise (Figure 1).

**Figure 2:**
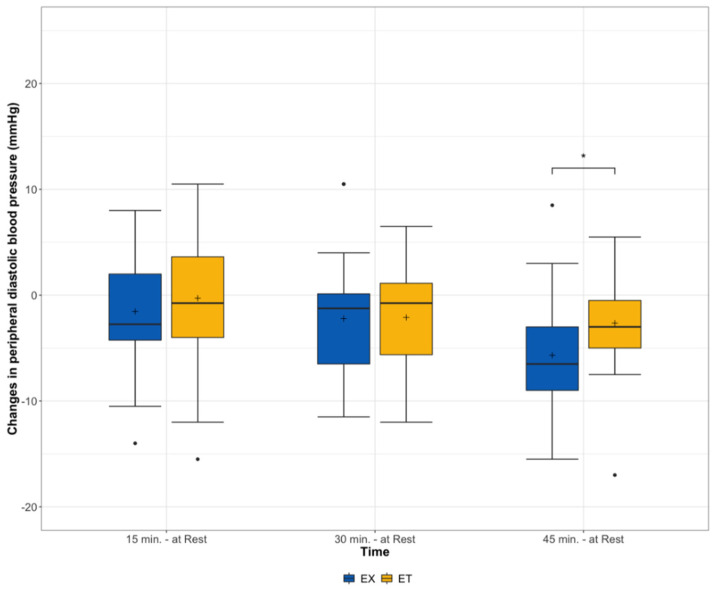
Changes in peripheral diastolic blood pressure from rest before and 15, 30, and 45 min after exercise stratified according to the type of exercise (ET = moderate endurance exercise, EX = exergaming session), interaction effect * = *p* < 0.05.

A significant difference in peripheral DBP between the two training procedures was shown 45 min after the training (*p* = 0.05) in favor of the EX. No significant interaction effects were found 15 min (*p* = 0.55) or 30 min (*p* = 0.93) after exercise (Figure 2).

Similar to the peripheral SBP, there was no change in central SBP after the ET (Table 2). The DBP was only slightly but statistically significantly reduced 45 min after the end of the training (Table 2). In contrast, after the EX, both the SBP and the DBP were still significantly reduced in the 45th minute compared to the pre-exercise BP (*p* < 0.001) (Table 2).

**Figure 3:**
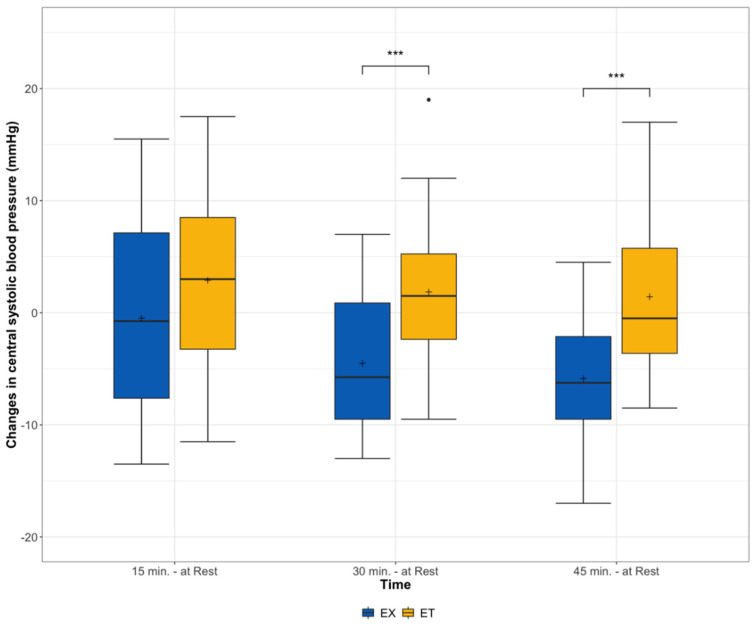
Changes in central systolic blood pressure from rest before and 15, 30, and 45 min after exercise stratified according to the type of exercise (ET = moderate endurance exercise, EX = exergaming session), Interaction effect *** = *p* < 0.001.

The interaction effects showed a tendency towards an advantage for the EX in central SBP 15 min after the exercise session (*p* = 0.07). Thirty minutes (*p* < 0.001) as well as 45 min (*p* < 0.001) after exercise, significant differences in favor of EX could be detected (Figure 3).

**Figure 4:**
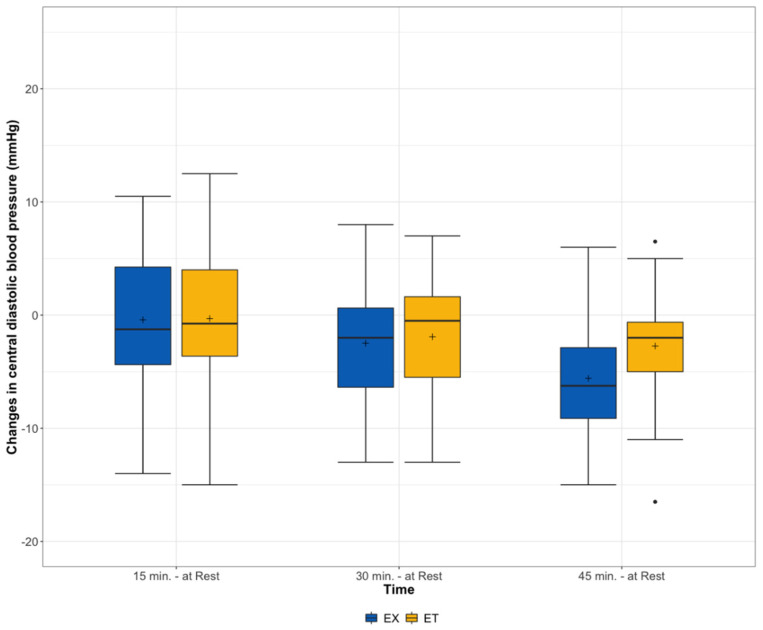
Changes in central diastolic blood pressure from rest before and 15, 30, and 45 min after exercise stratified according to the type of exercise (ET = moderate endurance exercise, EX = exergaming session).

In central DBP, the difference between the two groups showed a trend (*p* = 0.08) in favor of the EX 45 min after exercise (Figure 4).

## 4. Discussion

This is the first study to show that a single EX can result in greater peripheral and central BP reductions in healthy adults than a single ET.

The effects of acute exercise on peripheral BP have been previously studied. Numerous trials have determined PEH after different training protocols that last up to several hours after training [20,21]. The reduction in peripheral BP after the EX is comparable or even greater in magnitude than other studies have revealed after different exercise protocols [3,22].

The only low reduction in peripheral BP after the ET is surprising and in contrast to other studies [3,23]. A reason for the low PEH-effect after the ET may be the relatively short exercise duration of only 35 min. However, Liu and colleagues [3] determined more pronounced BP reductions after only 30 min of endurance exercise. Nevertheless, the exercise intensity in this study was higher (65% of VO_2_max), and their participants were prehypertensive sedentary individuals. Similarly, Pescatello et al. [24] determined stronger PEH effects after 30 min of endurance exercise compared to our ET when measuring BP five hours after exercise. However, they also included high normal to stage 1 hypertension participants. Only normotensive participants were included in the present study, which could account for the minor response, as baseline BP is reported as a predictor of post-exercise hypotension (PEH) [24]. In a more recent study, Pierce and colleagues [25] concluded that endurance training seems inferior to other exercise modes concerning PEH, which corresponds to our results.

Regarding central BP, the EX induced a similar reduction as seen in previous studies assessing moderate endurance or high-intensity interval training [23,26]. The non-invasive measurement of the central, aortic blood pressure is becoming increasingly important for the pathogenesis of cardiovascular diseases and the better characterization of different forms of arterial hypertension [27]. The central BP reflects the afterload of the heart and correlates with the myocardial oxygen consumption. Accordingly, the prognostic significance of central BP is evaluated higher than that of peripheral BP [14].

In the present study, post-exercise central SBP and DBP were significantly lower after the EX compared to the ET. The difference between peripheral and central SBP was the same after both exercise sessions compared to the pre-exercise baseline. This means that the relative physiological increase in vascular stiffness from central to peripheral was unchanged by both exercise types. This contrasts with the study of Goeder et al. [28], who found a greater decrease in central compared to peripheral BP after maximal exercise. Thus, it seems that changes in central BP are intensity-dependent.

The significant PEH of the EX may be attributed to the high exercise intensity. It is acknowledged that one of the causes of PEH is persistent shear stress-induced vasodilation that is not matched by increases in cardiac output [5,29]. The higher exercise intensity during the EX possibly led to a higher oxygen demand in the working muscle that consecutively provoked a greater blood flow through the vessel and, therefore, promoted greater shear-stress induced nitric oxide (NO) release [30].

Another possible explanation of the different BP reactions between the two exercise modes could be an exercise intensity induced alteration in baroreceptor function with subsequent sympathetic inhibition [24]. The influence of exercise intensity on the magnitude of PEH is supported by the literature [24,31].

Furthermore, a higher fluid loss via sweat could be debated as a reason for the difference in BP reaction. In the present study, the participants lost slightly more weight (−0.39 ± 0.27 kg) during the EX than during the ET (−0.30 ± 0.41 kg), indicating a higher fluid loss. However, the difference was marginal and not significant.

Another reason for the greater decrease in BP response after the EX may be the functional holistic training that provokes multi-joint movements engaging larger muscle groups. In a meta-analysis, Casonatto and colleagues [32] revealed, that resistance training sessions involving large muscle groups prompted greater PEH than sessions involving only small muscle groups. One of the physiological mechanisms that could explain the influence of muscle mass on BP after exercise may be a higher release in vasoactive substances (nitric oxide, prostaglandins) caused by a higher increase in blood flow [33].

To our knowledge, there is only one comparable study assessing the PEH effect of exergaming on peripheral BP in adults. Alves da Cruz et al. [34] found non-significant BP responses between an exergame session and a general cardiovascular rehabilitation program consisting of whole-body exercises for the upper and lower limbs and endurance exercise on a treadmill. In a study in children, Mills and colleagues [35] found that particularly high-intensity exergames, in contrast to exergames with lower intensity, exerted beneficial effects on flow-mediated dilatation.

Different studies have assessed the effects of regular exergaming on BP. McBain et al. [36] analyzed the effects of a self-designed high-intensity interval boxing exergame. After six weeks of intervention (3/week), the scholars found no apparent beneficial effects on BP compared to the control group, despite favorable changes in VO_2_max. De Carvalho and colleagues [37] compared seven weeks of regular (3/week) exergaming (Wii, Nintendo, Kyoto, Japan) with regular stretching exercise and observed an insignificant trend toward reduced systolic pressure in the group participating in the regular exergaming sessions. In a study conducted by Staiano et al. [38], a 24-week (3/week) home-based exergaming intervention improved systolic and diastolic BP, total cholesterol, and LDL-cholesterol in overweight and obese children. However, this trial used exergaming as one tool within a broader behavior change intervention.

Current literature does not allow a clear conclusion regarding the effectiveness of exergames on hemodynamic parameters. However, based on the present results, it seems that exergames, such as the ExerCube, which apply a sound training concept inducing high exercise intensities, have the potential to result in relevant physiological adaptions.

The effectiveness of the ExerCube may be attributed to the high exercise intensity and the functional holistic training that provokes multi-joint movements engaging larger muscle groups. Since the ExerCube tracks the HR throughout the game and adapts the game challenge and speed according to a predefined target HR, it can guarantee an adequate stimulus throughout the training session. Furthermore, the game requires the player to perform whole-body movements, which are precisely tracked by the motion-capturing system. Thus cheating, as often reported for hand-held motion sensors [39], is not possible.

Our findings suggest that the EX provided a more potent physiological stimulus compared to the selected ET. This is of relevance as the magnitude of BP reduction after acute exercise highly correlates with the magnitude of BP reduction after chronic training interventions [3]. Therefore, it can be assumed that regular exergaming may result in long-term BP reductions.

Thus, heart rate controlled exergames, such as the ExerCube, can present a promising training tool in cardiovascular rehabilitation and prevention. Since a lack of time and motivation is often stated as one of the barriers to regular exercise participation, exergames can potentially serve as an enjoyable and effective alternative to traditional ET. Previous studies have shown that the immersive and playful design of the ExerCube leads to higher exercise enjoyment than regular exercise programs [12,13,40]. This may enhance long-term training adherence.

Nevertheless, the superior effects of the EX may only apply to the young and healthy adults enrolled in the present study. Even though PEH has been reported in both young and older adults as well as in hypertensive and normotensive persons [22], the relevance of exercise intensity on PEH in different populations is not clear. A recent systematic review [41] revealed similar changes in BP after moderate endurance training and high-intensity interval training in older adults.

The game setup of the ExerCube could serve as a model of good practice for videogame designers. This could facilitate the development of exergames that not only target an appealing gaming experience but also integrate an effective training program. This can help expand the intervention approaches in cardiovascular prevention and rehabilitation.

### Limitations

There are several limitations that must be addressed. First, a relatively small sample size of healthy young participants was recruited, thus the results cannot be extrapolated to other populations. Further studies, including a larger sample size and different populations, should be carried out. However, it is expected that the BP reductions would be greater in individuals with hypertension compared to our mainly normotensive participants [42]. Since the exercise intensity in the ExerCube is individually tailored, it can be assumed that it allows an adequate exercise stimulus for different target groups.

Additionally, one could argue that a ramp-wise incremental GXT is superior for determining HRpeak. However, we chose the typical stepwise test to guarantee a steady-state for the lactate assessment.

Furthermore, the results are limited to the specific ExerCube protocol applied in the present study. Other exergames, especially ones inducing lower exercise intensities, will probably lead to different effects.

A further limitation is the difference in the exercise intensity between the ET and the EX. As the exercise intensity reached during the training in the ExerCube was not clear beforehand, a general endurance exercise protocol that was proven to modulate PEH was chosen. Future studies should compare exercise protocols of similar intensities and designs.

Finally, we only investigated the effects of an acute bout of EX. Further investigations assessing the long-term effects of EX are warranted and may reveal whether the acute effects could be accumulated.

## 5. Conclusions

It can be concluded that despite a lower time commitment, the EX is superior to an ET with regard to PEH. Since acute BP responses highly correlate with long-term changes in hemodynamic parameters, the ExerCube may present a time-efficient and motivating exercise approach for the prevention and rehabilitation of cardiovascular diseases. However, further studies assessing the effects of regular training in the ExerCube in different populations are highly warranted.

## Figures and Tables

**Table 1:** Participants’ characteristics.

Outcome	Mean ± SD
Age (y)	24.8 ± 3.9
Gender (f/m)	13/15
Body mass (kg)	68.9 ± 10.7
Height (cm)	171.9 ± 9.7
Body-Mass-Index (kg/m^2^)	23.2 ± 2.3
Waist-to-height-ratio	0.4 ± 0.1
HRmax (bpm)	194.1 ± 7.7

Abbreviations: SD = standard deviation; HRmax = maximal heart rate during graded exercise test; bpm = beats per minute.

**Table 2:** Peripheral and central SBP and DBP at rest before and 15, 30, and 45 min after exercise.

	Exergaming Session	*p*-Value *	Moderate Endurance Exercise	*p*-Value *
Peripheral SBP (mmHg)				
At rest (reference)	119.8 (115.1, 129.8)		118.8 (111.3, 124.6)	
15 min	122.5 (107.0, 129.5)	*p* = 0.615	119.5 (107.0, 128.3)	*p* = 0.969
30 min	116.5 (107.8, 121.5)	*p* < 0.001	118.0 (110.5, 124.3)	*p* = 0.544
45 min	113.5 (106.0, 119.0)	*p* < 0.001	117.0 (111.8, 122.3)	*p* = 0.137
Peripheral DBP (mmHg)				
At rest (reference)	69.8 (66.0, 74.1)		67.8 (64.4, 74.3)	
15 min	68.5 (62.5, 73.3)	*p* = 0.158	70.0 (63.8, 74.3)	*p* = 0.985
30 min	69.5 (61.0, 73.0)	*p* = 0.028	68.0 (61.8, 71.0)	*p* = 0.060
45 min	65.0 (59.8, 69.3)	*p* < 0.001	67.0 (61.0, 72.0)	*p* = 0.003
Central SBP (mmHg)				
At rest (reference)	109.8 (103.5, 120.1)		107.3 (102.8, 113.5)	
15 min	110.0 (100.0, 119.3)	*p* = 0.701	111.0 (99.0, 119.0)	*p* = 0.083
30 min	109.5 (99.3, 113.0)	*p* = 0.001	112.5 (100.0, 118.0)	*p* = 0.195
45 min	104.0 (97.8, 112.8)	*p* < 0.001	109.5 (102.0, 114.3)	*p* = 0.648
Central DBP (mmHg)				
At rest (reference)	71.3 (67.1, 75.0)		69.0 (65.4, 75.0)	
15 min	72.0 (63.0, 77.3)	*p* = 0.175	70.0 (64.5, 74.3)	*p* = 0.995
30 min	70.0 (62.8, 75.0)	*p* = 0.024	69.0 (62.0, 72.3)	*p* = 0.102
45 min	66.0 (60.5, 70,3)	*p* < 0.001	67.5 (61.0, 70.3)	*p* = 0.006

* Exact Wilcoxon test—comparison with resting value (reference). Abbreviations: SBP = systolic blood pressure; DBP = diastolic blood pressure. The variables were shown as the median and 1st and 3rd quartile.

## Data Availability

The data presented in this study are available on request from the corresponding author.
